# Is socioeconomic status associated with utilization of health care services in a single-payer universal health care system?

**DOI:** 10.1186/s12939-014-0115-1

**Published:** 2014-11-28

**Authors:** Dani Filc, Nadav Davidovich, Lena Novack, Ran D Balicer

**Affiliations:** Department of Politics and Government, Ben-Gurion University, POB 653, Beer Sheva, 8410501 Israel; Department of Health Systems Management, Ben-Gurion University, POB 653, Beer Sheva, 8410501 Israel; Department of Public Health, Ben-Gurion University, POB 653, Beer Sheva, 8410501 Israel; Clalit Research Institute, Chief Physician Office, Clalit Health Services, Arlozorov 101, Tel Aviv, Israel

## Abstract

**Objectives:**

To assess an association of Socio-economic status with utilization of health care services between years 2002 and 2008 in Israel.

**Methods:**

We retrospectively analyzed the utilization of health care services in a cohort of 100,000 members, 21 years and older, of a Clalit Health Services. The research compared utilization according to the neighborhood SES status; and clinic’s location as another SES proxy. Data included: Charlson Score morbidity factor, utilization of health care services (visits to primary physicians and specialists, purchase of pharmaceuticals, number of hospitalization days, visits to ED, utilization of laboratory tests and imaging). The analysis was performed using Generalized Linear Model (GLM) technique.

**Results:**

People with lower SES visited more the ED and primary physicians and were hospitalized for longer periods. People with higher SES visited more specialists, bought more prescription drugs and used more medical imaging. The associations between SES and most of the services we analyzed did not change between 2002 and 2008. However, the gap between lower and higher SES levels in ED visits and the use of prescription drugs slightly increased over time, while the gap in visits to specialists decreased.

**Conclusions:**

The research shows that even in a universal health care system SES is associated with utilization of health care services. In order to improve equity in utilization of services the Israeli public health should reduce economic barriers and in parallel invest in making information accessible to improve “navigation skills” for all.

Much attention has been drawn since the 1990s to inequality in health, especially to the relationship between Socio-Economical Status (SES) and health status [1-6]. While the main causes of those differences are the various social determinants of health, differences in access to health care services also explain health disparities. A significant corpus of research shows that SES affects patterns of utilization of health care services. The influence of health care services’ utilization on health inequalities is obviously greater in countries where health care services are mostly privatized. Yet, there is empirical evidence that even in countries with a developed public health care sector, SES influences utilization of health care services.

However, the evidence for inequalities in utilization of various health care services is contradictory, with a few studies showing that the influence of SES in accessing public health care is not consistent [7,8]. There are studies that show that lower SES populations encounter obstacles in accessing health care services [9,10]. On the other hand, several studies show that in countries where access to primary care and hospitalization are relatively free of charge, people with lower SES visit more GPs and are hospitalized more than people with higher SES [11-20], while populations from higher SES visits more specialists, makes more use of imaging diagnostic methods and has more access to more sophisticated therapies [18-25].

Since the association of SES with an access to the different health care services has important policy consequences, especially in countries with public, universal, health care systems, it is important to elucidate the different ways in which differences in SES affect the utilization of both ambulatory and hospital services. Moreover, since health care systems in most OECD countries) are undergoing processes of partial commodification and strengthening of market mechanisms such as co-payments, it is important to assess the evolution of the relationship between SES and health care utilization, since inequalities may increase over time, as found by Curtis and MacMinn [15] in the Canadian case.

In order to contribute to the research on the effects of SES on the utilization of health care services the present research studied the relationship between SES and utilization of health care services in Israel, as measured by objective computerized utilization data.

In Israel there are relatively few studies assessing the relationship between SES and utilization of health care services, and their findings are contradictory. Ellencweig et al. [26] and Neumark et al. [27] did not find a relationship between SES and utilization of services. Levine et al. [28] found that heavy smoking correlated with utilization of health care services in the army, but they did not find a statistically significant relationship between SES and utilization of health care services. On the other hand, Baron-Epel et al. [29] did find an association but only for Jewish citizens. These few studies were not based on objective data but on self-assessment of utilization obtained in interviews, nor evaluated whether the relationship between SES and health care utilization changed over time.

## Methods

### Objectives

The present research assesses an association of SES with utilization of health care services between years 2002 and 2008, a period in which the public financing of the national health expenditure decreased from 37.4% to 31.5% and the private share increased from 35.1% to 40.5%. The study evaluates a) whether in a single-payer health care system such as the Israeli one, SES is associated with utilization of health care services, b) whether the ongoing privatization of services’ financing increased inequality in utilization of health care services. To account for a possibility that SES is differentially associated with utilization of the diverse types of health care services, the research assessed this association separately for primary care physicians, specialists, visits to the ED, medical imaging, laboratory services, purchasing of prescription drugs and hospitalization. The present research was conducted using data from Clalit Health Services (CHS) computerized database. CHS is the largest public health fund in Israel and insures and provides care for about 56% of the Israeli population.

### Study design

We retrospectively analyzed the utilization of health care services in a cohort of 100,000 members of CHS between 2002 and 2008.

### Study population

The subjects were randomly chosen and were included in the study if they were 21 years and older by 2002 and remained registered in Clalit Health Services through 2008.

### Data collected

The data received from CHS computerized database included socio-demographic information as of 2002 (e.g., gender, age at enrollment, immigration status, complementary insurance status, social security subsidies, and the socio-economic status of the subject, as explained later in the text), Charlson comorbidity index (a score predicting the ten-year mortality based on a range of 22 comorbid conditions) and utilization of health care services (annual number of visits to primary physicians and specialists, purchase of pharmaceuticals, number of hospitalization days, visits to ED, utilization of laboratory tests and imaging). The research used an ecological parameter, the neighborhood SES score established by the Central Bureau of Statistics (CBS) as a proxy for SES status, represented by a Z-score comparing the Israeli neighborhood mean socio-economic status with the neighborhood of a subject. The SES status of a neighborhood is established based on multiple parameters collected by CBS for every small area, referred as “statistical region”. The list of factors utilized for the SES estimation includes: averaged number of subjects in a household, number of cars and computers in a household, average income, number of years of education, proportion with high school education and above, unemployment rate in the area, rate of unemployment within women, employment at minimal wage, etc. All these parameters have been recently collected at the last census in 2008 and continue to be updated annually based on CBS estimations. Each statistical neighborhood is assigned a score on a scale of 0-20, and further transformed into a Z-score enabling comparison of each statistical area to others in the country. A neighborhood is assigned to a subject based on his/her address, and is usually undefined for about 20% of the population due to technical problems of tracking population migration. In addition, we used the clinic’s location as another SES proxy, following CHS’s practice, by which patients usually attend their neighborhood clinic. This SES measurement was available for almost all subjects. Throughout the analysis we used an individual SES index obtained for each subject based on his/her address, which was grouped into 3 groups - low, medium and high - corresponding - to SES index percentiles <33.3%, 33.3-67.7%, and >67.7%. This measurement was completed with the categorical estimates of SES based on the clinic address for subjects without a verified address.

The survey was approved by the Ethical Committee of CHS.

### Statistical analysis

Categorical variables were presented as absolute numbers and as a proportion from the total number of observations. Continuous variables were described by average, standard deviation, median, minimum and maximum. To avoid exposure of sensitive data belonging to CHS, all parameters related to services utility were presented graphically as a proportion of the total average in 2002.

Comparisons between groups were performed using Chi-Square test and Fisher exact test where appropriate, whereas continuous variables were compared using *t*-test or ANOVA for normally distributed variables and Mann-Whitney and Kruskal-Wallis tests – for variables where normal distribution assumption did not hold.

All the outcomes were Poisson distributed, but due to the over-dispersion of the values – Negative-Binomial distribution was chosen for the outcome distribution in the model. The analysis was performed using Generalized Linear Model (GLM) technique (PROC GENMODE procedure).

The effect of socio-economic status (SES) over the period of 2002-2008 was tested by interaction between these two factors within the format of repeated measurements in GLM model and outcomes characterized by Negative-Binomial distribution and non pre-specified covariance matrix (defined as “unstructured” in procedure). The interaction between the SES and time was defined as significant when the main effects and the interaction term were found significant in the model. The effect of independent factors was expressed as Relative Risk (RR), which presented a multiplicative impact on the outcome variable and was obtained as an exponent of the regression coefficients in the final models. Statistical analysis was performed on Statistical Analysis Software (SAS) 9.2.

## Results

The study population consisted of 100,000 Clalit Health Services members whose average age by 2002 was 48.4 years and 46.4% men (Table 1). The population varied by its origin, with only 57.0% born in Israel, 16.6% in African or Asian countries, 13.2% originating from the former Soviet Union (FSU) and 10.6% born in USA or Europe. Every ninth subject belonged to the recent wave of immigration, primarily coming from the FSU. The study population represents a relatively healthy population with an average Charlson Score equal to 1.3 and median 0. More than half of the population (55.9%) was insured by a complementary insurance package in addition to the compulsory basic medical insurance.

**Table 1:** **Socio-demographical and medical characteristics by combined SES score**

**Subjects characteristics**	**Low SES score** ^**1**^ **N = 33,664**	**Medium SES score** ^**1**^ **N = 36,942**	**High SES score** ^**1**^ **N = 28,369**
Age by 2002, years			
Mean ± SD	44.99 ± 17.21	49.36 ± 17.91	51.23 ± 18.42
Median	42.00	48.00	50.00
Male gender	46.9%	46.6%	45.7%
Born in Israel	67.0%	49.5%	54.5%
USA, Europe	3.5%	11.1%	18.3%
Former Soviet Union	12.5%	16.9%	9.7%
Africa, Asia	14.7%	19.4%	15.4%
South America	0.5%	1.5%	1.7%
Ethiopia	1.9%	1.6%	0.3%
Immigrated after 1989	11.6%	14.3%	6.8%
Charlson score with age			
Mean ± SD	1.02 ± 1.68	1.37 ± 1.91	1.54 ± 2.00
Median	0.00	1.00	1.00
Charlson score without age			
Mean ± SD (n)	0.30 ± 0.85	0.40 ± 1.00	0.42 ± 1.02
Median	0.00	0.00	0.00
Covered by complimentary insurance	42.4%	57.8%	69.6%
SES index^2^			
Mean ± SD (n)	-0.84 ± 0.22	-0.00 ± 0.22	1.10 ± 0.48
Median	-0.90	0.00	0.99

^1^The SES index was grouped into 3 groups, low, medium and high corresponding to SES index percentiles <33.3%, 33.3-67.7%, and >67.7%.

^2^This factor is missing for 20.7% of the study population.

Due to the large sample all differences were found to be statistically significant.

The SES status measured by a Z-score could be verified for almost 80% of the study population (79.2%) with mean equal to Z = 0.06 and median 0 as compared to the national mean, with minimal score -2.24 and maximal score 3.37.

Table 1 shows demographical characteristics compared by SES status of the subjects. It shows that high SES status is associated with increased age, USA or European origin, and subjects who did not belong to the recent immigration wave of 1989, as well as higher coverage by a complementary insurance. On the other hand, high SES was also characterized by slightly higher Charlson morbidity index, corresponding to their increased age.

In 2002 the subjects in the study population had on average 0.2 visits to an Emergency Department (ED) during the year (i.e., one out of 5 subjects visited and ED in a year), and those who were hospitalized spent on average eight days of hospitalization per year. Naturally, hospitalizations in rehabilitations centers were rare, with only 511 patients hospitalized in 2002 with an average length of stay 38.9 days. The study population visited their primary care physician 3.6 times on average and half of them visited their physician at least once. Visits to specialists not requiring a referral were less frequent with 2 times on average to specialists in orthopedics, ophthalmologists, dermatologists, ear-throat and nose specialists and gynecologists and around one time to all other specialists who pre-requisite a referral from a primary physician.

**Figure 1:**
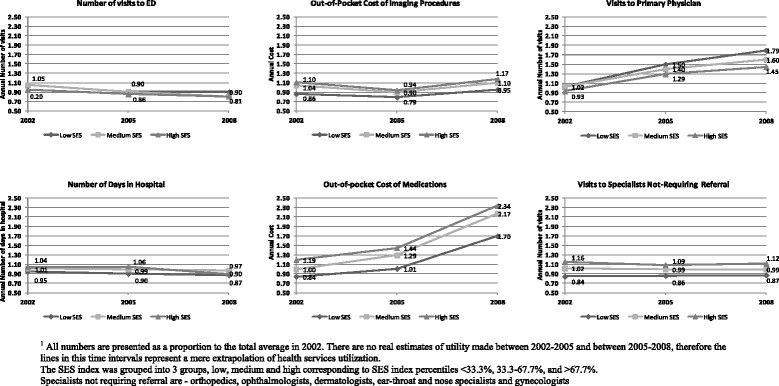
**Time trends in health services utility over the period 2002-2008, by SES** ^**1**^ **.**

Figure 1 shows the time trends in health services utility by SES, over the period of 2002-2008. Table 2 shows the results of the multivariable analysis of all primary endpoints health services utilization in 2008.

**Table 2:** **Multivariable analysis of primary endpoints in 2008**

	**ED visits**		**Hospitalization days**		**Cost of imaging**		**Medication cost**		**Visits to primary physician**		**Visits to specialists in hospital out-patient clinic**		**Visits to specialists without referral**	
**Parameter** ^**3**^	**RR** ^**2**^	**p-value**	**RR** ^**2**^	**p-value**	**RR** ^**2**^	**p-value**	**RR** ^**2**^	**p-value**	**RR** ^**2**^	**p-value**	**RR** ^**2**^	**p-value**	**RR** ^**2**^	**p-value**
Age by 2002, year	1.00	0.295	1.04	<.001	1.00	<.001	1.02	<.001	1.01	<.001	1.01	<.001	1.00	0.071
Male Gender	1.13	<.001	1.02	0.612	0.68	<.001	1.03	0.062	0.79	<.001	0.79	<.001	0.51	<.001
Origin vs Israeli born														
USA or Europe	0.98	0.582	0.89	0.067	1.04	0.389	1.07	0.008	0.90	<.001	1.02	0.706	1.02	0.217
Former USSR	0.78	<.001	0.98	0.748	1.03	0.442	1.17	<.001	0.89	<.001	0.91	0.046	1.04	0.009
Africa or Asia	1.23	<.001	1.24	<.001	1.16	<.001	0.96	0.045	1.11	<.001	1.32	<.001	1.12	<.001
South America	0.81	0.023	0.79	0.141	1.15	0.163	1.03	0.691	1.08	0.024	1.05	0.702	1.12	0.013
Ethiopia	0.79	0.008	1.16	0.324	0.81	0.026	2.43	<.001	1.02	0.583	0.70	0.010	0.96	0.389
Charlson score^1^	1.08	<.001	1.34	<.001	1.11	<.001	1.46	<.001	1.03	<.001	1.08	<.001	0.98	0.001
Complementary insurance	1.22	<.001	1.09	0.023	1.46	<.001	1.54	<.001	1.25	<.001	1.33	<.001	1.44	<.001
SES status (low, medium, high)	0.90	<.001	0.85	<.001	1.03	0.047	1.06	<.001	0.84	<.001	1.05	0.008	1.06	<.001

^1^Age is not included in the score.

^2^RR = Relative Risk obtained as exponent (Regression Coefficient (B)), presenting a multiplicative effect of an independent factor on the outcome variable.

^3^All independent factors are adjusted to all other factors in the table.

### Visits to the ED

The univariate analysis showed a slight difference between the three SES groups, with the lowest SES group visiting more the ED than the highest one. This finding was confirmed Relative Risk equal 1.11 (p-value < 0.001) for lower SES groups obtained in the multivariate analysis.

### Visits to primary physicians

Both univariate and multivariate analyses showed that people with lower SES visit more primary physicians than people with higher SES (RR = 1.19, p-value < 0.001).

### Visits to specialists

The univariate analysis showed that people with higher SES visit more specialists, and this difference exists for the three categories of specialists we checked - specialists in the ambulatory sector that can be visited without a family physician’s referral^a^, specialists in the ambulatory sector that require a referral and specialists in hospitals’ outpatient clinics. The multivariate analysis confirmed that higher SES subjects visited specialists more frequently, specifically the number of visits to specialists in subjects in SES higher levels would increase 1.05-fold compared to lower levels (p-value = 0.008).

### Prescription drugs

Both univariate and multivariate analysis showed a clear relationship of SES with the purchasing of prescription drugs, with higher SES consuming more medicaments, reflected in a 1.06 increase (p-value < 0.001).

### Medical imaging

Higher SES was found as a predictor of higher utilization of MRI and CT (RR = 1.03, p-value = 0.047).

### Hospitalizations

The univariate model (for years 2002 and 2005) showed that people from higher SES spent more days hospitalized than people in the lowest SES group. When controlling for age and other variables in the multivariate model we found that, lower SES is a positive predictor of hospitalization, i.e., the number of hospitalization days increased 1.18-folds for representatives of lower SES levels, compared to higher levels (p-value < 0.001).

### Changes over time

The associations between SES and most of the services we analyzed did not change between 2002 and 2008. However, the gap between lower and higher SES levels in ED visits and the use of prescription drugs slightly increased over time, while the gap in visits to specialists decreased over the period covered by the research.

## Discussion

The 1994 National Health Insurance bill was supposed to promote both horizontal (for those in the same need) and vertical (those who have different needs) equity in access to health care services [30]. Our present study shows, however, that SES is associated with an access to services not only in private health care systems. Even in a universal, single-payer health care system, SES still affects utilization of health care services. In some cases, the magnitude of an association has even enhanced over time. In accord with previous research on a possible impact of SES on utilization of health care services, our study shows that people with lower SES visit more the primary physician, ED and are hospitalized for longer time, while people belonging to a higher SES group visit more specialists, make more use of medical imaging and purchase more prescription drugs than people in lower SES groups [19,21,24,25,31-33]. These findings are especially significant since not as most previous studies, our research is based on data of actual utilization rather than on self-report.

There are three main pathways that explain these findings. Firstly, several studies show that people of lower SES suffer from poorer health [6,34]. Poorer health may explain why people with lower SES make more use of the point of entrance to the health care system (the primary physician) and spend more days in hospital (where more serious conditions are treated). This is the standard explanation in the literature for the fact that, when the system is mostly public, people with lower SES make more use of primary physicians. Studies also show that people in lower SES groups have a greater probability to be hospitalized for “ambulatory care sensitive conditions”, i.e. conditions for which adequate ambulatory care would have prevented hospitalization [19]. It should be noted that in our sample the Charlson index was almost similar for the three groups (as a matter of fact, it was slightly higher for the highest SES group, probably reflecting the older average age within this group), meaning that differences in health status alone cannot explain the differences in utilization of health care services. However, in a similar previous study, Shadmi et al. [35], showed that using the Adjusted Clinical Groups indicator of morbidity instead of the Charlson index, shows that morbidity burden does offer an explanation of differential use of services. The second pathway is related to the fact that people in higher SES are more able to navigate the system, thus being capable to access the more sophisticated services (in our study expressed by specialists and medical imaging utilization) and reduce acute health care situations expressed in ED referral and hospitalizations.

The third pathway is related to the ability to pay for services. In the Israeli health care systems there are no copayments for primary care visits and hospitalizations, nor individual ceilings for hospitalization costs. On the other side, specialists’ visits, medical imaging and prescription drugs require copayments. The latter are relatively high and have increased during the years covered by the present study. A survey conducted by the Myers-JDC-Brookdale Institute in 2006 showed that more than 25% of the people in Israel’s lowest quintile report that during the last year they refrained from buying prescription drugs or visiting a specialist because of the financial burden it represented [36].

An important contribution of our study is the analysis of patterns of utilization through time. This evolution of differences in utilization over time is of special interest. Our data show that the SES is associated with number of visits to the ED increased between 2002 and 2008, similarly to the purchase of prescription drugs. Contrarily, the gap for visits to specialists (both in the community and at outpatient hospital clinics) decreased along time. It is difficult to point to a single explanation for these findings. The increasing magnitude of association of SES with the purchase of prescription drugs is probably related to the significant rise in drug copayments, as governments increased co-payments in order to shift part of the health care costs from the public sector to individual users. A possible explanation to the diminishing association of SES with frequency of visits to specialists at the same period of time, might be that people in the higher SES purchase more private insurance, and thus tend to visit private specialists paid by this insurance instead of specialists in the public health care system. Moreover, maybe there was an improvement in the abilities of lower SES groups in navigating the system. In order to verify these assumptions, further research should be done to better assess the causes underlying the differences in the modification of utilization patterns through time.

### Strengths and weaknesses

The main strengths of our research are the fact that we were able to analyze data on actual utilization and not self-reported utilization, that the analysis assessed the utilization of several and varied services (primary care, specialists, hospitalization, prescription drugs and medical imaging), and the longitudinal dimension of the research, allowing to estimate the changes over time of the relationship between SES and utilization of health care services. The main weakness of the study is the use of an ecological measurement of SES adscription as opposed to individual data. Even though this is a method amply used in the literature studying the relationship between SES and utilization of health care services [37-39], ecological studies may lack sensitivity for illuminating the ways in which SES influences utilization of health care services.

Another weakness stems from the fact that individual address is not defined for 20.7% of the sample in the Central Bureau of Statistics' data. Thus, we have based our classification into three SES groups on the address of the subject’s clinic.

The inclusion criterion limiting the study to Clalit members who stayed insured during the follow-up period 2002-2008 implies a certain selection bias, since lower SES groups are overrepresented among the latter. However, while the possibility of selection bias limits the generalization of the findings to all the sick funds, the magnitude of Clalit and the size of the sample make the association between SES and utilization of services important from a policy perspective. The patients’ younger age in the study population (due to the minima follow-up of 6 years required as an inclusion criterion), could bring to less variability in outcomes. However, with an actual wide range in age in the study (SD = 18 years), this scenario is unlikely to happen because of the exclusion criterion.

In the current report the authors could not present absolute numbers, in order to avoid an exposure of sensitive data, which limits the transparency of the results, however does not affect their validity.

## Conclusions

Even in a public health care system SES is associated with utilization of health care services. Our results show a consistent pattern of different utilization of health care services according to SES, with lower SES patients using more the ED and being more hospitalized and patients from higher SES using more ambulatory services (especially specialists’ services) and consuming more prescription drugs. Most of these patterns persisted throughout the investigated period but, against our original assumption, only the relationship of low SES and low utilization of prescription drugs deepened through time. In our research it is not clear whether lower SES population’s poorer health contributed to the SES gap. While the fact that they were hospitalized for longer periods hints to more severe conditions, the Charlson index did not confirm that the lower SES population has a worse health status. The causes of these trends demand further research, however, we can put forward two assumptions. First, that copayments are negatively associated with equity in utilization of health care services, since people with lower SES use less of those services or goods requiring copayments (pharmaceuticals, medical imaging and specialists), and since the SES gap for medication increased over time, as co-payments grew. Second, that knowledge on how to navigate the system is related to the utilization of specialists and medical imaging (for the relationship between copayments and inequality in utilization of services see [40,41]. For the relationship between utilization and “navigation skills” (see [42]). Thus, reducing economic barriers such as copayments and investing in making information accessible and improving “navigation skills” for all, could improve equity in the utilization of services in the Israeli public health system.

## Endnote

^a^There are five specialists that can be accessed without a referral: specialists in orthopedics, ophthalmologists, dermatologists, ear-throat and nose specialists and gynecologists.
